# Baseline liver function tests and full blood count indices and their association with progression of chronic kidney disease and renal outcomes in Aboriginal and Torres Strait Islander people: the eGFR follow- up study

**DOI:** 10.1186/s12882-020-02185-x

**Published:** 2020-12-01

**Authors:** Sandawana William Majoni, Federica Barzi, Wendy Hoy, Richard J. MacIsaac, Alan Cass, Louise Maple-Brown, Jaquelyne T. Hughes

**Affiliations:** 1 Department of Nephrology, Division of Medicine, Royal Darwin Hospital, P.O. Box 41326, Casuarina, Darwin, Northern Territory Australia; 2 Flinders University and Northern Territory Medical Program, Royal Darwin Hospital Campus, Darwin, Australia; 3 Wellbeing and Preventable Chronic Diseases, Menzies School of Health Research, Charles Darwin University, Darwin, Australia; 4 Centre for Chronic Disease, The University of Queensland, Brisbane, St Lucia Australia; 5 Baker Heart and Diabetes Institute, Melbourne, Australia; 6 Department of Medicine, University of Melbourne, Melbourne, Australia; 7 St. Vincent’s Hospital Melbourne, Melbourne, Australia; 8 Department of Endocrinology & Diabetes, St Vincent’s Hospital Melbourne and The University of Melbourne, Fitzroy, Victoria Australia

**Keywords:** Aboriginal and Torres Strait islander Australians, Estimated glomerular filtration rate, Liver function tests, Full blood count indices, Renal outcomes

## Abstract

**Background:**

Determination of risks for chronic kidney disease (CKD) progression could improve strategies to reduce progression to ESKD. The eGFR Study recruited a cohort of adult Aboriginal and Torres Strait Islander people (Indigenous Australians) from Northern Queensland, Northern Territory and Western Australia, aiming to address the heavy CKD burden experienced within these communities.

**Methods:**

Using data from the eGFR study, we explored the association of baseline liver function tests (LFTs) (alanine aminotransferase (ALT), alkaline phosphatase (ALP), gamma-glutamyl transpeptidase (GGT), bilirubin and albumin) and full blood count (FBC) indices (white blood cell and red blood cell counts and haemoglobin) with annual eGFR decline and renal outcomes (first of 30% decline in eGFR with a follow-up eGFR < 60 mL/min/1.73 m^2^, initiation of renal replacement therapy, or renal death). Comparisons of baseline variables across eGFR categories were calculated using analysis of variance and logistic regression as appropriate. Linear and multivariable regression models were used to estimate the annual change in eGFR for changes in FBC indices and LFTs. Cox proportional hazard models were used to estimate the hazard ratio for developing renal outcome for changes in baseline FBC indices and LFTs.

**Results:**

Of 547 participants, 540 had at least one baseline measure of LFTs and FBC indices. The mean age was 46.1 (14.7) years and 63.6% were female. The median follow-up was 3.1 (IQR 2.8–3.6) years. Annual decline in eGFR was associated with low serum albumin (*p* < 0.001) and haemoglobin (*p* = 0.007). After adjustment for age, gender, urine albumin/creatinine ratio, diabetes, BMI, CRP, WHR, alcohol consumption, cholesterol and triglycerides, low serum albumin (*p* < 0.001), haemoglobin (*p* = 0.012) and bilirubin (*p* = 0.011) were associated with annual decline in eGFR.

Renal outcomes were inversely associated with serum albumin (p < 0.001), bilirubin (p = 0.012) and haemoglobin (p < 0.001) and directly with GGT (p = 0.007) and ALP (p < 0.001). Other FBC indices and LFTs were not associated with annual decline in eGFR or renal outcomes.

**Conclusions:**

GGT, ALP, bilirubin, albumin and haemoglobin independently associate with renal outcomes. Contrary to findings from other studies, no association was found between renal outcomes and other FBC indices. These findings may help focus strategies to prevent disease progression in this high-risk population.

**Supplementary Information:**

The online version contains supplementary material available at 10.1186/s12882-020-02185-x.

## Background

Aboriginal and Torres Strait Islander people (the First Nation people of Australia/ Indigenous Australians) suffer some of the highest rates of end stage kidney disease (ESKD) [1]. Clear determination of the potential risks for decline in estimated glomerular filtration rate (eGFR) could increase understanding and improve the ability to implement targeted strategies to reduce the progression to ESKD. The association of biomarkers of inflammation with kidney function decline has been documented in several studies [2, 3] and also reported in the eGFR Study [4]. Some recent studies have shown possible association of elevated concentrations of white blood cells with kidney function decline [5, 6] and others have shown that the neutrophil/lymphocyte ratio, a marker of systemic inflammation, may be associated with poor renal outcomes [7, 8]. Furthermore, there is also emerging evidence of an association of abnormal liver function tests with decline in eGFR and increasing albuminuria [9].

The eGFR Study was a longitudinal study of 654 Indigenous Australian adults from more than 20 sites in urban, regional, and remote Australian regions known to have high incidence of ESKD, and participants were selected across five strata of health, diabetes status, and kidney function [10–12]. This study confirmed the accuracy of the CKD-EPI equation in estimating GFR in Indigenous Australians [12]. In a longitudinal follow-up, progression of CKD occurred rapidly, with loss of kidney function at approximately three times higher rate than expected through healthy ageing [13, 14]. Predictors of progression of CKD included elevated baseline urine albumin/creatinine ratio (UACR) and C-reactive protein (CRP) concentrations [9, 14, 15]. The inflammatory marker, soluble tumour necrosis factor receptor 1 (sTNFR1), was also associated with CKD progression, where this outcome was independent of albuminuria and eGFR in participants with diabetes [4]. Serum bilirubin concentration was positively associated with haemoglobin and total cholesterol; however there was an inverse association with UACR [9]. Cardiometabolic risk factors were not strong predictors for eGFR decline in Indigenous Australians who had normoalbuminuria at baseline [16].

An assessment of the potential association of abnormal liver function tests (LFTs) and full blood count (FBC) indices with annual eGFR decline in the eGFR Study will therefore increase our understanding of their potential role as predictors of eGFR decline in Indigenous Australians. We therefore undertook an exploratory study of whether abnormal baseline concentrations of liver function tests (LFTS- i.e. high alanine aminotransferase (ALT), gamma-glutamyl transpeptidase (GGT), aspartate aminotransferase (AST), and bilirubin and low albumin) and FBC indices (high white blood cell (WBC) counts and red blood cell (RBC) counts and low haemoglobin (Hb)), were associated with annual decline in eGFR and renal outcomes.

## Methods

### Study design, participants, and data source

Participants were recruited from 20 community sites within five large geographic regions: Top End of the Northern Territory of Australia, Central Australia, Far North Queensland (North Queensland and the Torres Strait), and the Kimberley and Goldfields regions in Western Australia. The details of the eGFR Study protocol have been described elsewhere [11].

Using an existing data set from the eGFR Study, an analysis was performed to explore the association of baseline concentrations of LFTs and FBC indices with annual eGFR decline and renal outcomes.

### Definitions of outcomes

The follow-up was the time from the date of baseline to the follow-up serum creatinine measurements (range 0.52–5.75 years). Outcomes were defined, as in previous published work from the eGFR Study [4, 13, 14, 16], as follows; 1) the annual eGFR decline as annual change in CKD-EPI eGFR ([CKD-EPI eGFR at follow up-CKD-EPI eGFR at baseline]/follow up period) and 2) the combined renal outcome defined as the first of the following: an absolute 30% decline in eGFR with a follow-up eGFR < 60 mL/min/1.73 m^2^, death from renal causes, or initiation of renal replacement therapy. All deaths occurring when eGFR declined to < 15 mL/min/1.73 m^2^ were defined as renal deaths. Participants were censored at the time the first end point was reached.

### Laboratory and clinical measurements

The laboratory and clinical measurements methods were described elsewhere in the previous work from the eGFR Study [4, 11, 13, 14, 16]. In summary, at baseline and follow-up, non-fasting venous blood samples were collected, and pathology and clinical records were reviewed. Serum creatinine, LFTs, FBC, CRP, UACR and other metabolite assays were performed at each centre as part of standard clinical care. At each centre, an accredited laboratory performed assaying of creatinine and the assay used had traceability to the Isotope Dilution Mass Spectrometry reference method [4]. Additionally, measurement of serum creatinine in all samples was performed at a central laboratory, using Roche enzymatic method on a Beckman- Coulter DxC 800 analyser (Fullerton, CA, USA).

### Statistical analysis

A descriptive analysis of the demographic, clinical, biochemical and outcome data summarising the data as mean and standard deviation (SD) for continuous normally distributed variables and median and inter-quartile range (IQR) for continuous variables with skewed distributions was performed. Comparisons of baseline variables across eGFR categories were carried out using analysis of variance for continuous variables and Chi-Squared tests for categorical variables, Univariable linear regression models were used to regress the annual change in eGFR on FBC indices and on LFTs. Multivariable regression models were used to examine the potential confounding effect on FBC indices and on LFTs of other factors shown to be associated with eGFR decline including age and gender, alcohol consumption, urine ACR, diabetes, CRP, body mass index, waist-hip ratio (WHR), cholesterol and triglycerides. Univariable and multivariable Cox proportional hazard models were used to estimate the hazard ratio (HR) for developing a combined renal outcome for changes in baseline FBC indices and concentrations of LFTs (AST, ALT, ALP, GGT, bilirubin and albumin). Proportional hazards (PH) assumptions were tested using scaled Schoenfeld residuals to assess the validity of the proportional hazards assumption. A plot of the scaled Schoenfeld residuals over time should have zero slope if the PH assumption was met. This was tested for each covariate, and for the whole model.

For model building, independent variables were transformed where indicated by the distribution of the variable. Potential effect modifications of CRP and diabetes were investigated with linear regression models and Cox proportional hazard models that included the interaction terms between CRP and measures of LFTs and FBC and between diabetes and measures of LFTs and FBC indices respectively. We excluded UACR in the adjustment for models examination the association between the outcomes and serum albumin concentrations due the correlation between serum albumin concentrations and UACR.

Sensitivity analyses were carried out where the regression analyses were performed with each of the predictor variables divided into quintiles.

Two-sided tests were used for all the analyses and the level of significance was set at *p* < 0.05. All analyses were performed using STATA software version 15.1 (StataCorp 2017©1985–2017 StataCorp LP).

## Results

### Available data on baseline concentrations of LFTs and FBC indices and baseline characteristics

**Fig. 1:**
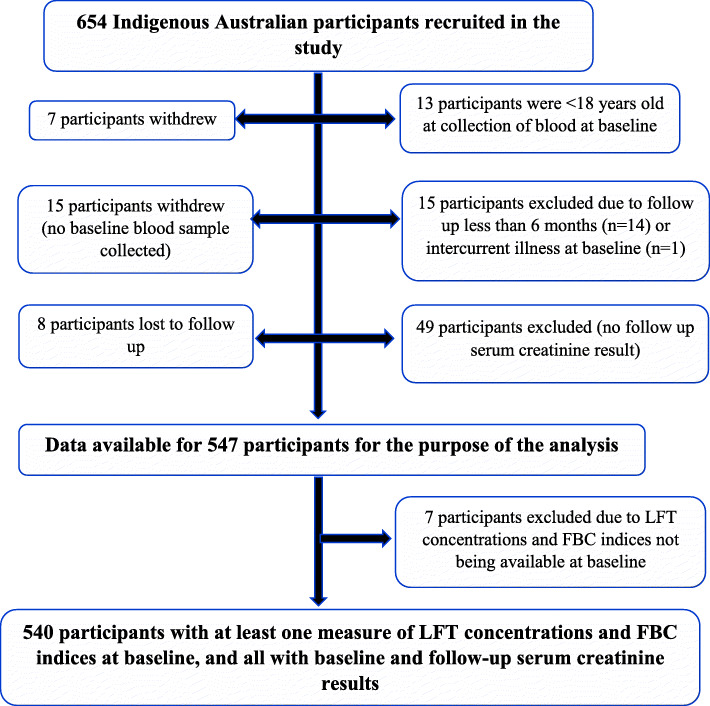
Participant data selection for analysis in the study and handling missing data. LFT, Liver function tests; FBC, Full blood count

Of the 654 Indigenous Australian participants in the baseline eGFR Study [15], data were available on 547 participants for the purposes of this study. Of these, 540 participants had at least one measure of LFTs concentrations and FBC indices at baseline (Fig. 1). There were no data on AST, differential WBC (for determination of neutrophil/lymphocyte ratio) and red blood cell distribution width in the dataset so analysis on these could not be performed.

The mean age was 46.1 (14.7) years and 348 (63.6%) participants were female. The median follow-up was 3.1 years (IQR 2.8 to 3.6 years). Mean annual change in CKD-EPI eGFR (95% confidence interval) for the analysis population was − 3.1 (− 3.6 to − 2.6) mL/min per 1.73m^2^. The baseline clinical characteristics stratified by eGFR are summarised in Table 1. At baseline, older age, the presence of diabetes, gender, low concentrations of ALT, albumin, bilirubin and haemoglobin and high concentrations of ALP, high UACR, systolic blood pressure, WHR and HbA1c were significantly associated with lower eGFR categories. There was no significant association between each of baseline concentrations of GGT and FBC indices and eGFR categories. (Table 1).

**Table 1:** Baseline clinical characteristics, measures of liver function, FBC and cardiometabolic markers stratified by eGFR

Baseline variable	eGFR (CKD EPI) < 60 ml/min per 1.73 m^**2**^ (***n*** = 83)	eGFR (CKD EPI) 60–89 ml/min per 1.73 m^**2**^ (***n*** = 110)	eGFR (CKD EPI) > 90 ml/min per 1.73 m^**2**^ (***n*** = 354)	***P*** Value*	Total (***N*** = -547)
Age (years)	58.5 (12.6)	51.1 (13.6)	41.6 (13.1)	< 0.001	46.1 (14.6)
Female n (%)	49 (59)	60 (54.5)	239 (67.1)	0.533	348 (63.6)
Diabetes n (%)	54 (65.1)	50 (45.6)	135 (38.1)	0.007	239 (43.7)
ALT (U/L)	23 (17.5–29.5))	27 (19–36)	26 (19–38)	0.005	26 (19–36)
GGT (U/L)	32 (23–77)	33 (22–50)	34 (25–58)	0.391	34 (24–61)
ALP (U/L)	121.5 (96–156.5)	95 (76–112)	93 (77–119)	< 0.001	96 (78–122)
ACR (mg/mmol)	53.7 (12.4–220.9)	1.6 (0.8–13.1)	1.5 (0.7–7.0)	< 0.001	2.1 (0.7–17.7)
CRP (mg/L)	5.0 (3.0–13.0)	5.0 (2.5–9.0)	6.0 (3.0–11.0)	0.646	5.8 (3–11)
Albumin (g/L)	39.4 (4.6)	42.3 (3.98)	42.6 (4.1)	< 0.001	42.1 (4.3)
Bilirubin (μmol/L)	5 (3–8)	7 (5–10)	7 (5–9)	0.002	6.5 (7.9–9.6)
WBC (×10^9^)	8.1 (3.5)	7.78 (3.3)	7.8 (3.2)	0.650	7.9 (3.2)
Hb (g/L)	124.1 (18.8)	135.5 (16.7)	139.3 (16.4)	< 0.001	136.3 (17.6)
RBC (× 10^12^)	4.1 (1.7)	4.7 (1.0)	4.6 (1.5)	0.060	4.6 (1.4)
SBP (mmHg)	123.0 (17.4)	120.2 (21.0)	116.5 (16.1)	0.002	118.2 (17.5)
DBP (mmHg)	74.5 (9.8)	74.5 (9.8)	74.8 (10.5)	0.712	74.7 (10.2)
WHR (0.1 unit)	1.0 (0.08)	0.9 (0.1)	0.9 (0.1)	< 0.001	0.9 (0.1)
HbA1c (%)	6.5 (5.9–8.4)	6.1 (5.7–7.2)	6.0 (5.6–7.0)	0.012	6.1 (5.6–7.3)
HbA1c (mmol/L)	47.5 (41.0–68.3)	43.2 (38.8–55.2)	42.1 (37.7–53.0)	0.012	43.2 (37.7–56.3)
Cholesterol (mmol/L)	4.5 (1.1)	4.8 (1.1)	4.9 (1.1)	0.012	4.8 (1.1)
Triglycerides (mmol/L)	2.2 (1.6–3.4)	1.8 (1.2–2.4)	1.8 (1.3–2.5)	0.069	1.8 (1.3–2.5)

Data are mean (SD) or median (25th, 75th percentile) unless otherwise specified. *CKD-EPI* CKD-Epidemiology Collaboration, *ALT* Alanine Aminotransferase, *GGT* Gamma-glutamyl transferase, *ALP* Alkaline phosphatase, *ACR* Albumin to creatinine ratio, *CRP* C-reactive protein, *FBC* Full Blood Count, *WBC* white blood cell count, *Hb* haemoglobin, *RBC* red blood cell count, *SBP* Systolic blood pressure, *DBP* diastolic blood pressure, *WHR* waist-hip ratio, *HbA1c* glycated haemoglobin, *U/L* units per litre, *g/mol* grams per mol, *g/L* grams per litre, *mmHg* millimetres of mercury, *mmol/L* millimoles per litre, *ng/L* nanograms per litre, *pg/L* picograms per litres. *p-value of comparisons across eGFR categories and was calculated using ANOVA for continuous variables and logistic regression for categorical variables

### Associations of baseline concentrations of LFTs and measures of FBC indices with annual decline in eGFR

Univariable analyses results showed no relationship between annual decline in eGFR and the following; log ALT (*p* = 0.315), log GGT (*p* = 0.224), log ALP (*p* = 0.226), log bilirubin (*p* = 0.599), WBC (*p* = 0.720), RBC (*p* = 0.483) and CRP (*p* = 0.827). A strong association was demonstrated between annual decline in eGFR and low concentrations of serum albumin (*P* < 0.001) and haemoglobin (*p* = 0.007). (Table 2).

**Table 2:** Regression models of the relationship between annual decline in eGFR and baseline concentrations of measures of liver function tests and full blood count indices

Baseline factor	unadjusted		Adjusted for age and gender		Adjusted for age, gender and UACR		Adjusted for age gender, UACR and diabetes		Adjusted for age, gender, UACR, diabetes, total cholesterol, triglycerides, BMI, WHR, Alcohol consumption	
	β (95% CI)	***p***-value	β (95%CI)	***p***-value	β (95% CI)	***p***-value	β (95% CI)	***p***-value	β (95% CI)	***p***-value
Log ALT (U/L)	0.55 (−0.52–1.62)	0.315	0.66 (− 0.45–1.77)	0.245	0.56 (− 0.54–1.67)	0.315	0.55 (− 0.57–1.67)	0.336	− 0.06 (−1.16–1.04)	0.914
Log GGT (U/L)	− 0.45 (−1.17–0.27)	0.224	− 0.39 (− 1.13–0.34)	0.295	−0.18 (− 0.92–0.56)	0.636	−0.07 (− 0.83–0.69)	0.857	−0.18 (− 0.94–0.59)	0.655
Log ALP (U/L)	− 0.98 (−2.58–0.61)	0.226	−1.05 (−2.65–0.56)	0.200	−0.04 (− 1.67–0.60)	0.966	0.12 (− 1.55–1.77)	0.897	0.38 (− 1.26–2.02)	0.639
Log Bilirubin (μmol/L)	− 0.27 (− 1.28–0.74)	0.599	−0.17 (− 1.21–0.86)	0.742	−1.00 (− 2.07–0.07)	0.067	−1.04 (− 2.13–0.06)	0.063	−1.43 (− 2.52- -0.33)	0.010
Serum albumin (g/L)	0.34 (0.22–0.46)	< 0.001	0.36 (0.24–0.49)	< 0.001	N/A	N/A	0.34 (0.21–0.47)^a^	< 0.001	0.31 (0.18–0.44)^a^	< 0.001
Hb (g/L)	0.04 (0.01–0.07)	0.007	0.06 (0.03–0.10)	0.001	0.05 (0.01–0.08)	0.014	0.04 (0.01–0.08)	0.018	0.013 (−0.02–0.05)	0.011
WBC (× 10^9^/L)	0.03 (−0.14–0.20)	0.720	0.03 (−0.14–0.20)	0.739	0.08 (− 0.09–0.25)	0.354	0.07 (− 0.10–0.24)	0.404	0.02 (− 0.15–0.19)	0.797
RBC (× 10^12^/L)	0.14 (− 0.24–0.51)	0.483	0.17 (− 0.22–0.56)	0.391	0.15 (− 0.24–0.54)	0.456	0.14 (− 0.26–0.53)	0.493	−0.04 (− 0.45–0.36)	0.832

^a^UACR no included in the model

*β* β-coefficient, *CI* Confidence Intervals, *ALT* Alanine Aminotransferase, *GGT* Gamma-glutamyl transferase, *ALP* Alkaline phosphatase, *UACR* Urine Albumin/creatinine ratio, *U/L* units per litre, *g/L* grams per litre, *μmol/L* micromoles per litre; cholesterol mmol/L, triglycerides mmol/L, C-reactive protein mg/

After adjustment for age, gender, UACR, diabetes and CRP, body mass index, waist-hip ratio (WHR), alcohol consumption, cholesterol and triglycerides, only low concentrations of serum albumin (*p* < 0.001) and haemoglobin (*p* = 0.012), and high concentrations of bilirubin (*p* = 0.011) were significantly associated with annual decline in eGFR. However, with this adjustment, there was no significant association between annual decline in eGFR and concentrations of the rest of measures of LFTs and FBC indices. (Table 2).

When divided into quintiles and adjusted for age, gender, UACR, diabetes and CRP, BMI, WHR, alcohol consumption, cholesterol and triglycerides, there was a positive linear association with more preserved eGFR across the five quintiles of concentrations of serum albumin, RBC, haemoglobin, relative to lowest (first quintile). (Tables S1 and S2).

### Association of baseline concentrations of measures of LFTs and FBC indices with the renal outcomes

Table 3 details the associations between baseline concentration of measures of LFTs and FBC indices with the combined renal outcome. 12.4% (*n* = 68) of the 540 participants experienced the outcome over the follow-up period of a median of 3.1 (IQR 2.8–3.6) years The following baseline measures were associated with reduced crude hazard ratio of the renal outcome; concentrations of serum ALT, serum albumin and haemoglobin. These associations were independent of age, gender, UACR, diabetes and CRP, body mass index, waist-hip ratio (WHR), cholesterol and triglycerides for serum bilirubin, albumin, and haemoglobin but not for concentration of ALT. Concentrations of GGT and ALP were associated with increased crude hazard ratio of renal outcomes. This increased hazard ratio persisted after adjustment for age, gender and UACR, diabetes, alcohol consumption, CRP, BMI, WHR, cholesterol and triglycerides.

**Table 3:** Cox regression models of association of measures of concentrations of liver function tests and full blood count indices with renal outcomes

Baseline measure	Crude		Adjusted for age and gender		Adjusted for age, gender and UACR		Adjusted for age gender, UACR and diabetes		Adjusted for age, gender, UACR, diabetes, CRP, total cholesterol, triglycerides, BMI, WHR, Alcohol consumption	
	HR (95% CI)	***p***-value	HR (95% CI)	***p***-value	HR (95% CI)	***p***-value	HR (95% CI)	***p***-value	HR (95% CI)	***p***-value
Log ALT (U/L)	0.58 (0.35–0.94)	0.027	0.67 (0.40–1.12)	0.129	0.64 (0.37–1.10)	0.109	0.54 (0.31–0.98)	0.041	0.64 (0.35–1.17)	0.147
Log GTT (U/L)	1.48 (1.10–2.00)	0.012	1.49 (1.10–2.02)	0.011	1.53 (1.10–2.13)	0.011	1.54 (1.08–2.16)	0.017	1.55 (1.13–2.14)	0.004
Log ALP (U/L)	11.38 (5.88–22.01)	< 0.001	13.40 (6.68–27.23)	< 0.001	9.35 (4.40–19.86)	< 0.001	7.56 (3.55–16.08)	< 0.001	4.95 (2.32–10.52)	< 0.001
Log Bilirubin (μmol/L)	0.42 (0.26–0.68)	< 0.001	0.35 (0.21–0.57)	< 0.001	0.52 (0.31–0.89)	0.016	0.49 (0.28–0.87)	0.015	0.62 (0.35–1.08)	0.011
Albumin (g/l)	0.87 (0.84–0.90)	< 0.001	0.88 (0.84–0.91)	< 0.001	N/A	N/A	0.86 (0.81–0.91)^a^	< 0.001	0.84 (0.79–0.90)^a^	< 0.001
WBC (×10^9^/L)	1.07 (0.99–1.15)	0.108	1.07 (1.00–1.15)	0.044	1.05 (0.97–1.14)	0.219	1.05 (0.97–1.13)	0.245	1.05 (0.96–1.13)	0.283
RBC (×10^12^/L)	0.93 (0.82–1.05)	0.237	0.94 (0.82–1.08)	0.392	0.96 (0.83–1.10)	0.545	0.96 (0.83–1.10)	0.535	0.96 (0.82–1.13)	0.622
Haemoglobin (g/L)	0.96 (0.95–0.97)	< 0.001	0.95 (0.94–0.97)	< 0.001	0.95 (0.94–0.97)	< 0.001	0.95 (0.94–0.97)	< 0.001	0.96 (0.94–0.98)	< 0.001

^a^UACR not include in the model

*HR* Hazard Ratio, *95% CI* 95% Confidence Intervals, *ALT* Alanine Aminotransferase, *GGT* Gamma-glutamyl transferase, *ALP* Alkaline phosphatase, *UACR* Urine albumin/creatinine ratio, *U/L* units per litre, *g/L* grams per litre, *μmol/L* micromoles per litre, cholesterol mmol/L, triglycerides mmol/L, C-reactive protein mg/L, *BMI* body mass index, *WHR* waist hip ratio

RBC and WBC were not associated with crude hazard ratio of renal outcomes. This lack of relationship with hazard ratio of renal outcomes remained on multivariable analyses.

There was no significant interaction between CRP and measures of LFTs and FBC indices and between diabetes and measures of LFTs and FBC indices with annual eGFR decline and renal outcomes, respectively.

## Discussion

In this analysis of the longitudinal eGFR Study, in a population at high risk of renal disease and progression to ESKD, we explored the association of measures of liver function and full blood count indices with annual decline in eGFR and combined renal outcomes.

For the first outcome, the annual decline in eGFR, we report an association with low concentrations of serum albumin, haemoglobin, and high concentrations of serum bilirubin.

For the second outcome, defined as the incidence of a combined renal end point, which was the first of the following: a 30% decline in eGFR with a follow-up eGFR < 60 mL/min/1.73 m^2^, death from renal causes, or initiation of renal replacement therapy, we report an inverse association with concentrations of serum albumin, serum bilirubin and haemoglobin and a positive association with concentrations of GGT and ALP.

Our study supports the emerging evidence that high bilirubin concentrations may be protective against decline in kidney function and poor renal outcomes. We have previously reported from the eGFR study the inverse association of log-bilirubin with UACR [9]. Several other studies suggest a protective role of high serum bilirubin against progression of chronic kidney disease and poor renal outcomes and this may be related to delaying progression of fibrosis-related kidney disease [17–22].

In our study of participants with high risk of CKD, there was a statistically significant decrease in the baseline concentrations of serum ALT across categories of decreasing eGFR. However, there was no significant relationship between baseline concentration of serum ALT and the annual decline in eGFR. The inverse association of ALT with the crude hazard of renal outcomes disappeared after adjustment for other covariates. To our knowledge, there are no previous studies exploring the association of serum concentrations of ALT and AST with renal outcomes. AST was not measured in this study. However, observational studies have described lower serum concentrations of serum aminotransferases over time in adults with CKD [23, 24], with multifactorial causes postulated. The same studies have suggested that new reference ranges may need to be set for these enzymes in people with CKD although the rationale for this is not clear from the current evidence [23, 24].

Our analysis has shown a statistically significant association between increased concentrations of serum GGT and poor renal outcomes although an association with annual decline in eGFR was lacking. Other studies have also suggested that GGT is an independent predictor of mortality in patients with stage 4–5 chronic kidney disease [25]. Some previous studies have suggested an association of serum GGT with development and progression of CKD [26–28] although some studies have suggested that in some ethnic groups this association is confounded by other factors such as body mass index, life style factors, lipids, smoking and heavy alcohol intake [27, 29]. This positive association of high baseline concentrations of GGT with poor renal outcomes may indicate that the serum levels of this enzyme can be used as a surrogate marker of risk for poor renal outcomes. The mechanism linking GGT and progression of CKD is unclear and will need to be further studied although oxidative stress and inflammation in synergy with serum ferritin have been suggested [26].

In this study, there was a positive association between serum ALP concentrations and increased risk of poor renal outcomes. Serum ALP concentrations increased with progression in CKD. However, this is likely to be the bone isoenzyme of the ALP (not liver) which increases with the onset and severity of renal bone disease [30–32]. Although this supports the potential use of serum ALP as predictor of poor outcomes in people with CKD [25, 31, 32], the liver isoenzyme and association with decline in eGFR and renal outcomes would need further studies.

As expected, and found in many studies, serum albumin concentrations were inversely associated with decline in eGFR and high risk of adverse renal outcomes [33–36]. Similarly, the inverse relationship between haemoglobin and decline in eGFR and poor renal outcomes is expected.

Our current study showed no association of concentrations of WBC and RBC with annual decline in eGFR and renal outcomes. The potential role of the neutrophil/lymphocyte ratio could not be assessed because these variables were not available among the data. Recent studies have demonstrated mixed results on the predictive role of WBC on decline of eGFR in the people with CKD. Some studies have shown that elevated WBC count was a strong predictor of kidney function decline [5], high monocyte count was significantly associated with risks of incident CKD and CKD progression to ESKD [6] and low WBC count was independently associated with CKD progression in the elderly [37]. Other studies have demonstrated the potential role of the high neutrophil/lymphocyte ratio as a predictor of poor renal outcomes [7, 8]. However, in a study of inflammatory markers including hsCRP, WBC count and ferritin, hsCRP and ferritin stratified by albumin associated with RRT and rapid renal progression, but WBC count was not associated with renal outcomes [38]. These mixed results suggest the need for further studies on this potential association of WBC with renal outcomes.

Our analysis showed no association between RBC counts and annual decline in eGFR and renal outcomes after adjusting for other covariates. In our study, we did not have data on red blood cell distribution width (RDW), a measure of the range of variation of RBC volume. The association of red blood cell count and progression of renal disease remains poorly understood. Most studies have demonstrated the shortened life span of red blood cells with progression of CKD [39–41]. Studies have also suggested the predictive value of RDW, for cardiovascular and risk of CKD and renal outcomes [42, 43]. Therefore, further studies of the association between renal outcomes and RBC counts and RDW are needed.

Since this is a cohort of high-risk CKD population, specifically adult Aboriginal and Torres Strait Islander people, we believe these findings may be generalized to all populations at high risk of CKD although further studies in other high risk populations are required.

### Limitations

There were some limitations to our study. There was no data on AST, viral hepatitis, and history of liver disease, which would need to be used in the adjustment for the potential role of the liver function tests (ALT, GGT, ALP, Albumin and bilirubin) in the Cox model. This will need further exploration as studies have indicated the association of liver disease with progression of CKD [44–46]. The absence of other parameters of the FBC indices, such as the differential WBC and red RDW, meant that we were unable to corroborate the details of the association of these indices with eGFR decline and renal outcomes.

The small number of participants in the study who reached the renal outcomes potentially limited the power to detect the associations and increased the lack of precision of estimates for the associations. The median follow-up of 3.1 years was relatively short, and the ongoing long-term follow-up will provide more robust assessment of these associations.

## Conclusion

Our findings show that measures of liver function (GGT, ALP, bilirubin and albumin) and haemoglobin, routinely measured in clinical practice, are independently associated with poor renal outcomes. Contrary to results from other studies, we did not find any association of FBC indices (WBC count and RBC count) with progression of CKD and renal outcomes. Noting the need for further studies for clarity, our findings may help focus strategies to prevent disease progression in this high-risk population.

## Supplementary Information

**Additional file 1: Table S1.** Association of quintiles of baseline concentrations of measures of LFTS and annual decline in eGFR adjusted for age, gender, UACR, diabetes and CRP, total cholesterol, triglycerides, BMI, WHR, Alcohol consumption. **Table S2.** Association of quintiles of levels of FBC indices and annual decline in eGFR adjusted for age, gender, UACR, diabetes and CRP, total cholesterol, triglycerides, BMI, WHR, Alcohol consumption.

## Data Availability

All data supporting the study are presented in the manuscript and available on a request to the eGFR study editorial committee.

## Abbreviations

ALT: Alanine aminotransferase
GGT: Gamma-glutamyl transferase
ALP: Alkaline phosphatase
WBC: White blood cell count
RBC: Red blood cell count
CKD: Chronic kidney disease
ESKD: End stage kidney disease
HR: Hazard ratio
FBC: Full Blood Count
UACR: Urine albumin to Creatinine ratio
CRP: C-Reactive protein
hsCRP: Highly sensitive C-Reactive protein
BMI: Body Mass Index
WHR: Waist hip ratio
PH: Proportional hazards

## References

[1] Hoy WE, Mott SA, Mc Donald SP (2016). An expanded nationwide view of chronic kidney disease in Aboriginal Australians. Nephrology (Carlton, Vic).

[2] Amdur RL, Feldman HI, Gupta J, Yang W, Kanetsky P, Shlipak M, Rahman M, Lash JP, Townsend RR, Ojo A (2016). Inflammation and progression of CKD: the CRIC study. Clin J Am Soc Nephrol.

[3] Gupta J, Mitra N, Kanetsky PA, Devaney J, Wing MR, Reilly M, Shah VO, Balakrishnan VS, Guzman NJ, Girndt M (2012). Association between albuminuria, kidney function, and inflammatory biomarker profile in CKD in CRIC. Clin J Am Soc Nephrol.

[4] Barr ELM, Barzi F, Hughes JT, Jerums G, Hoy WE, O'Dea K, Jones GRD, Lawton PD, Brown ADH, Thomas M (2018). High baseline levels of tumor necrosis factor receptor 1 are associated with progression of kidney disease in indigenous Australians with diabetes: the eGFR follow-up study. Diabetes Care.

[5] Fan F, Jia J, Li J, Huo Y, Zhang Y (2017). White blood cell count predicts the odds of kidney function decline in a Chinese community-based population. BMC Nephrol.

[6] Bowe B, Xie Y, Xian H, Li T, Al-Aly Z (2017). Association between monocyte count and risk of incident CKD and progression to ESRD. Clin J Am Soc Nephrol.

[7] Yoshitomi R, Nakayama M, Sakoh T, Fukui A, Katafuchi E, Seki M, Tsuda S, Nakano T, Tsuruya K, Kitazono T (2019). High neutrophil/lymphocyte ratio is associated with poor renal outcomes in Japanese patients with chronic kidney disease. Ren Fail.

[8] Yuan Q, Wang J, Peng Z, Zhou Q, Xiao X, Xie Y, Wang W, Huang L, Tang W, Sun D (2019). Neutrophil-to-lymphocyte ratio and incident end-stage renal disease in Chinese patients with chronic kidney disease: results from the Chinese cohort study of chronic kidney disease (C-STRIDE). J Transl Med.

[9] Hughes JT, Barzi F, Hoy WE, Jones GRD, Rathnayake G, Majoni SW, Thomas MAB, Sinha A, Cass A, MacIsaac RJ (2017). Bilirubin concentration is positively associated with haemoglobin concentration and inversely associated with albumin to creatinine ratio among indigenous Australians: eGFR study. Clin Biochem.

[10] Maple-Brown LJ, Hughes JT, Lawton PD, Jones GR, Ellis AG, Drabsch K, Brown AD, Cass A, Hoy WE, MacIsaac RJ (2012). Accurate assessment of kidney function in indigenous Australians: the estimated GFR study. Am J Kidney Dis.

[11] Maple-Brown LJ, Lawton PD, Hughes JT, Sharma SK, Jones GR, Ellis AG, Hoy W, Cass A, Macisaac RJ, Sinha AK (2010). Study protocol--accurate assessment of kidney function in indigenous Australians: aims and methods of the eGFR study. BMC Public Health.

[12] Maple-Brown LJ, Ekinci EI, Hughes JT, Chatfield M, Lawton PD, Jones GR, Ellis AG, Sinha A, Cass A, Hoy WE (2014). Performance of formulas for estimating glomerular filtration rate in indigenous Australians with and without type 2 diabetes: the eGFR study. Diabetic Med.

[13] Maple-Brown LJ, Hughes JT, Ritte R, Barzi F, Hoy WE, Lawton PD, Jones GRD, Death E, Simmonds A, Sinha AK (2016). Progression of kidney disease in indigenous Australians: the eGFR follow-up study. Clin J Am Soc Nephrol.

[14] Barzi F, Jones GRD, Hughes JT, Lawton PD, Hoy W, O'Dea K, Jerums G, MacIsaac RJ, Cass A, Maple-Brown LJ (2018). Trajectories of eGFR decline over a four year period in an indigenous Australian population at high risk of CKD-the eGFR follow up study. Clin Biochem.

[15] Ekinci EI, Hughes JT, Chatfield MD, Lawton PD, Jones GR, Ellis AG, Cass A, Thomas M, MacIsaac RJ, O'Dea K (2015). Hyperfiltration in indigenous Australians with and without diabetes. Nephrol Dialysis Transplantation.

[16] Barr EL, Barzi F, Hughes JT, Jerums G, O'Dea K, Brown AD, Ekinci EI, Jones GR, Lawton PD, Sinha A (2018). Contribution of cardiometabolic risk factors to estimated glomerular filtration rate decline in Indigenous Australians with and without albuminuria - the eGFR Follow-up Study. Nephrology (Carlton, Vic).

[17] Park S, Kim DH, Hwang JH, Kim Y-C, Kim JH, Lim CS, Kim YS, Yang SH, Lee JP (2017). Elevated bilirubin levels are associated with a better renal prognosis and ameliorate kidney fibrosis. PLoS One.

[18] Sakoh T, Nakayama M, Tanaka S, Yoshitomi R, Ura Y, Nishimoto H, Fukui A, Shikuwa Y, Tsuruya K, Kitazono T (2015). Association of serum total bilirubin with renal outcome in Japanese patients with stages 3–5 chronic kidney disease. Metab Clin Exp.

[19] Yang TL, Lin YC, Lin YC, Huang CY, Chen HH, Wu MS (2017). Total Bilirubin in Prognosis for Mortality in End‐Stage Renal Disease Patients on Peritoneal Dialysis Therapy. J Am Heart Assoc.

[20] Boon AC, Bulmer AC, Coombes JS, Fassett RG (2014). Circulating bilirubin and defense against kidney disease and cardiovascular mortality: mechanisms contributing to protection in clinical investigations. Am J Physiol Renal Physiol.

[21] Li M, Li X, Liu Y, Liu X, Song Y, Zhao J, Mohan C, Wu T, Peng A, Qin L (2018). Relationship between serum bilirubin levels s and the progression of renal function in patients with chronic kidney disease and hyperuricemia. Clin Chim Acta.

[22] Liu Y, Li M, Song Y, Liu X, Zhao J, Deng B, Peng A, Qin L (2018). Association of serum bilirubin with renal outcomes in Han Chinese patients with chronic kidney disease. Clin Chim Acta.

[23] Ray L, Nanda SK, Chatterjee A, Sarangi R, Ganguly S (2015). A comparative study of serum aminotransferases in chronic kidney disease with and without end-stage renal disease: need for new reference ranges. Int J Appl Basic Med Res.

[24] Sette LHBC, EPD AL (2014). Liver enzymes serum levels in patients with chronic kidney disease on hemodialysis: a comprehensive review. Clinics (Sao Paulo).

[25] Caravaca-Fontán F, Azevedo L, Bayo M, Gonzales-Candia B, Luna E, Caravaca F (2017). High levels of both serum gamma-glutamyl transferase and alkaline phosphatase are independent preictors of mortality in patients with stage 4-5 chronic kidney disease. Nefrologia.

[26] Chen T, Ren Y, Gao Y, Tian H (2017). Serum gamma-Glutamyl Transferase and ferritin synergistically associated with the rate of chronic kidney disease. Dis Markers.

[27] Noborisaka Y, Ishizaki M, Yamazaki M, Honda R, Yamada Y (2013). Elevated serum gamma-Glutamyltransferase (GGT) activity and the development of chronic kidney disease (CKD) in cigarette smokers. Nephrourol Mon.

[28] Lee DY, Han K, Yu JH, Park S, Heo JI, Seo JA, Kim NH, Yoo HJ, Kim SG, Kim SM (2020). Gamma-glutamyl transferase variability can predict the development of end-stage of renal disease: a nationwide population-based study. Sci Rep.

[29] Kunutsor SK, Laukkanen JA (2017). Gamma-glutamyltransferase and risk of chronic kidney disease: a prospective cohort study. Clin Chim Acta.

[30] Kalantar-Zadeh K, Molnar MZ, Kovesdy CP, Mucsi I, Bunnapradist S (2004). Management of mineral and bone disorder after kidney transplantation. Curr Opinion Nephrol Hypertension..

[31] Taliercio JJ, Schold JD, Simon JF, Arrigain S, Tang A, Saab G, Nally JV, Navaneethan SD (2013). Prognostic importance of serum alkaline phosphatase in CKD stages 3-4 in a clinical population. Am J kidney Dis.

[32] Kovesdy CP, Ureche V, Lu JL, Kalantar-Zadeh K (2010). Outcome predictability of serum alkaline phosphatase in men with pre-dialysis CKD. Nephrol Dialysis Transplant.

[33] Haller C (2005). Hypoalbuminemia in renal failure: pathogenesis and therapeutic considerations. Kidney Blood Pressure Res.

[34] Alves FC, Sun J, Qureshi AR, Dai L, Snaedal S, Bárány P, Heimbürger O, Lindholm B, Stenvinkel P (2018). The higher mortality associated with low serum albumin is dependent on systemic inflammation in end-stage kidney disease. PLoS One.

[35] Lang J, Katz R, Ix JH, Gutierrez OM, Peralta CA, Parikh CR, Satterfield S, Petrovic S, Devarajan P, Bennett M (2017). Association of serum albumin levels with kidney function decline and incident chronic kidney disease in elders. Nephrology Dialysis Transplantation.

[36] Zhang J, Zhang R, Wang Y, Li H, Han Q, Wu Y, Wang T, Liu F (2019). The level of serum albumin is associated with renal prognosis in patients with diabetic nephropathy. J Diabetes Res.

[37] Arai Y, Kanda E, Iimori S, Naito S, Noda Y, Sasaki S, Sohara E, Okado T, Rai T, Uchida S (2018). Low white blood cell count is independently associated with chronic kidney disease progression in the elderly: the CKD-ROUTE study. Clin Exp Nephrol.

[38] Tsai Y-C, Hung C-C, Kuo M-C, Tsai J-C, Yeh S-M, Hwang S-J, Chiu Y-W, Kuo H-T, Chang J-M, Chen H-C (2013). Association of hsCRP, white blood cell count and ferritin with renal outcome in chronic kidney disease patients. PLoS One.

[39] Li JH, Luo JF, Jiang Y, Ma YJ, Ji YQ, Zhu GL, Zhou C, Chu HW, Zhang HD (2019). Red blood cell lifespan shortening in patients with early-stage chronic kidney disease. Kidney Blood Pressure Res.

[40] Ly J, Marticorena R, Donnelly S (2004). Red blood cell survival in chronic renal failure. Am J Kidney Dis.

[41] Sato Y, Mizuguchi T, Shigenaga S, Yoshikawa E, Chujo K, Minakuchi J, Kawashima S (2012). Shortened red blood cell lifespan is related to the dose of erythropoiesis-stimulating agents requirement in patients on hemodialysis. Therapeutic Apheresis Dialysis.

[42] Zhang M, Zhang Y, Li C, He L (2015). Association between red blood cell distribution and renal function in patients with untreated type 2 diabetes mellitus. Ren Fail.

[43] Zhang J, Cao J, Nie W, Shen H, Hui X (2018). Red cell distribution width is an independent risk factor of patients with renal function damage in type 1 diabetes mellitus of children in China. Ann Clin Lab Sci.

[44] Chen C-Y, Lin C-J, Lin C-S, Sun F-J, Pan C-F, Chen H-H, Wu C-J (2017). The prevalence and association of chronic kidney disease and diabetes in liver cirrhosis using different estimated glomerular filtration rate equation. Oncotarget.

[45] Jang HR, Kang D, Sinn DH, Gu S, Cho SJ, Lee JE, Huh W, Paik SW, Ryu S, Chang Y (2018). Nonalcoholic fatty liver disease accelerates kidney function decline in patients with chronic kidney disease: a cohort study. Sci Rep.

[46] Kiapidou S, Liava C, Kalogirou M, Akriviadis E, Sinakos E (2019). Chronic kidney disease in patients with non-alcoholic fatty liver disease: what the Hepatologist should know?. Ann Hepatol..

