# Supplementary material for: Baseline liver function tests and full blood count indices and their association with progression of chronic kidney disease and renal outcomes in Aboriginal and Torres Strait Islander people: the eGFR follow- up study
Source: BMC Nephrol. 2020 Dec 1;21:523. doi: 10.1186/s12882-020-02185-x (PMC7709437; doi:10.1186/s12882-020-02185-x)
Supplement: Supplementary file 1 — Additional file 1: Table S1. Association of quintiles of baseline concentrations of measures of LFTS and annual decline in eGFR adjusted for age, gender, UACR, diabetes and CRP, total cholesterol, triglycerides, BMI, WHR, Alcohol consumption. Table S2. Association of quintiles of levels of FBC indices and annual decline in eGFR adjusted for age, gender, UACR, diabetes and CRP, total cholesterol, triglycerides, BMI, WHR, Alcohol consumption. [file 12882_2020_2185_MOESM1_ESM.docx]

**Table S1: Association of quintiles of baseline concentrations of measures of LFTS and annual decline in eGFR adjusted for age, gender, UACR,** **diabetes and CRP, total cholesterol, triglycerides, BMI, WHR, Alcohol consumption**

| **Quintiles** | **β-coefficient (95% CI)** | | | | | | | | | |
| --- | --- | --- | --- | --- | --- | --- | --- | --- | --- | --- |
|  | **ALT** | | **GGT** | | **ALP** | | **Bilirubin** | | **albumin^a^** | |
|  |  | **p-value** |  | **p-value** |  | **p-value** |  | **p-value** |  | **p-value** |
| 1 (reference) |  |  |  |  |  |  |  |  |  |  |
| 2 | 0.90 (-0.72-2.5) | 0.275 | 0.31 (-1.33-1.96) | 0.709 | 0.25 (-1.43-1.93) | 0.767 | -0.59 (-2.11-0.92) | 0.442 | 1.69 (0.08-3.30) | 0.039 |
| 3 | -0.13 (-1.91-1.66) | 0.888 | 0.43 (-1.25-2.10) | 0.618 | 0.08 (-1.56-1.73) | 0.920 | 1.19 (-0.77- 3.16) | 0.233 | 1.47 (-0.13-3.08) | 0.072 |
| 4 | 0.43 (-1.27-2.14) | 0.617 | 0.72 (-.93-2.37) | 0.390 | 1.27 (-0.42-2.97) | 0.142 | -0.13 (-1.74-1.48) | 0.873 | 2.89 (1.18-4.59) | 0.001 |
| 5 | 1.44 (-0.31-3.19) | 0.107 | -0.66 (-2.33-1.02) | 0.441 | 0.05 (-1.66-1.78) | 0.951 | -1.43 (-3.11-0.25) | 0.095 | 2.93 (1.25-4.61) | 0.001 |
| CI, confidence intervals; ALT, alanine aminotransferase; GGT, Gamma-glutamyl transferase; ALP, Alkaline phosphatase, ^a^UACR excluded for models | | | | | | | | | | |

**Table S2: Association of quintiles of levels of FBC indices and annual decline in eGFR adjusted for age, gender, UACR, diabetes and CRP, total cholesterol, triglycerides, BMI, WHR, Alcohol consumption**

| **Quintiles** | **β-coefficient (95% CI)** | | | | | |
| --- | --- | --- | --- | --- | --- | --- |
|  | **WBC** | | **RBC** | | **haemoglobin** | |
|  |  | **p-value** |  | **p-value** |  | **p-value** |
| 1 (reference) |  |  |  |  |  |  |
| 2 | 0.26 (-1.46-1.98) | 0.767 | 1.51 (-0.18-3.20) | 0.079 | 1.32 (-0.41-3.05) | 0.134 |
| 3 | 0.03 (-1.67-1.74) | 0.972 | 1.23 (-0.62-3.08) | 0.191 | 2.25 (0.53-3.98) | 0.011 |
| 4 | 0.98 (-0.73-2.69) | 0.260 | 1.27 (-0.54-3.09) | 0.170 | 1.32 (-0.49-3.12) | 0.010 |
| 5 | 1.09 (-0.63-2.82) | 0.584 | 1.99 (0.11-3.86) | 0.038 | 3.17 (1.22-5.12) | 0.001 |
| CI, confidence interval; WBC, white blood cell count; RBC, red blood cell count. | | | | | | |
